# The locus of impairment in English developmental letter position dyslexia

**DOI:** 10.3389/fnhum.2014.00356

**Published:** 2014-06-03

**Authors:** Yvette Kezilas, Saskia Kohnen, Meredith McKague, Anne Castles

**Affiliations:** ^1^Department of Cognitive Science, ARC Centre of Excellence in Cognition and its Disorders, Macquarie UniversitySydney, NSW, Australia; ^2^Melbourne School of Psychological Science, The University of Melbourne, Melbourne, VIC, Australia

**Keywords:** phonological output deficit, orthographic input lexicon deficit, orthographic-visual analysis deficit, migration errors, substitution errors, developmental dyslexia

## Abstract

Many children with reading difficulties display phonological deficits and struggle to acquire non-lexical reading skills. However, not all children with reading difficulties have these problems, such as children with selective letter position dyslexia (LPD), who make excessive migration errors (such as reading *slime* as “smile”). Previous research has explored three possible loci for the deficit – the phonological output buffer, the orthographic input lexicon, and the orthographic-visual analysis stage of reading. While there is compelling evidence against a phonological output buffer and orthographic input lexicon deficit account of English LPD, the evidence in support of an orthographic-visual analysis deficit is currently limited. In this multiple single-case study with three English-speaking children with developmental LPD, we aimed to both replicate and extend previous findings regarding the locus of impairment in English LPD. First, we ruled out a phonological output buffer and an orthographic input lexicon deficit by administering tasks that directly assess phonological processing and lexical guessing. We then went on to directly assess whether or not children with LPD have an orthographic-visual analysis deficit by modifying two tasks that have previously been used to localize processing at this level: a same-different decision task and a non-word reading task. The results from these tasks indicate that LPD is most likely caused by a deficit specific to the coding of letter positions at the orthographic-visual analysis stage of reading. These findings provide further evidence for the heterogeneity of dyslexia and its underlying causes.

## INTRODUCTION

The last three decades have seen an emphasis on the role that impaired phonological processing plays in developmental dyslexia. Various researchers have posited that at the core of dyslexia lies an impairment in the ability to represent, store, and retrieve speech sounds (Stanovich, 1988; Snowling, 1998, 2001; Ramus, 2003). This phonological deficit is proposed to be linked to the difficulty children with dyslexia experience in learning the mappings between letters and speech sounds, which is often remediated using phonics training (see Castles et al., 2009; McArthur et al., 2012). The phonological deficit account of dyslexia is supported by a multitude of correlational, longitudinal, and training studies that have found developmental dyslexia to typically be associated with poor phonological awareness (e.g., Høien et al., 1989), slow lexical retrieval skills (e.g., Denckla and Rudel, 1976), and poor verbal short-term memory (e.g., Mann et al., 1980; Mann and Liberman, 1984).

However, not all children with dyslexia have a phonological impairment. For example, children with surface dyslexia appear to have no difficulties with mapping letters onto speech sounds, as is evidenced by their ability to read non-words as proficiently as their peers (e.g., Castles and Coltheart, 1993; Broom and Doctor, 1995; Castles and Coltheart, 1996; Temple, 1997). Instead, surface dyslexics have been thought to have problems with orthographic processing, resulting in excessive reading errors where an irregular word is sounded out incorrectly using common letter-sound rules (e.g., *yacht* is read as if it rhymed with *matched*). The existence of cases of developmental dyslexia where phonological processing appears intact suggests that while some dyslexias may be attributed to an impairment in phonological processing, other dyslexias are not. Here, we provide further evidence for the heterogeneity of dyslexia and its underlying causes by furthering the investigation of the locus of impairment in English-speaking children with developmental letter position dyslexia (LPD).

The hallmark symptom of LPD is an elevated tendency to make “migration errors,” where the order of letters within migratable words (more commonly known as anagrams) is confused, resulting in the misreading of a word as its migration partner (e.g., *slime* is read as “smile”). While migration errors are frequently made by beginning readers (Kohnen and Castles, 2013), English children with LPD have been found to make up to four times the number of migration errors made by their peers (Kohnen et al., 2012). Children with LPD have particularly high migration error rates when reading words where the transposition of letters in the middle of a word can lead to another word (e.g., *slime–smile, diary–dairy*). Intriguingly, cases of selective LPD have been documented, where all other reading processes appear intact (Friedmann and Rahamim, 2007; Kohnen et al., 2012). Children with selective LPD read as accurately and as fluently as their peers – except when they are asked to read migratable words.

**FIGURE 1:**
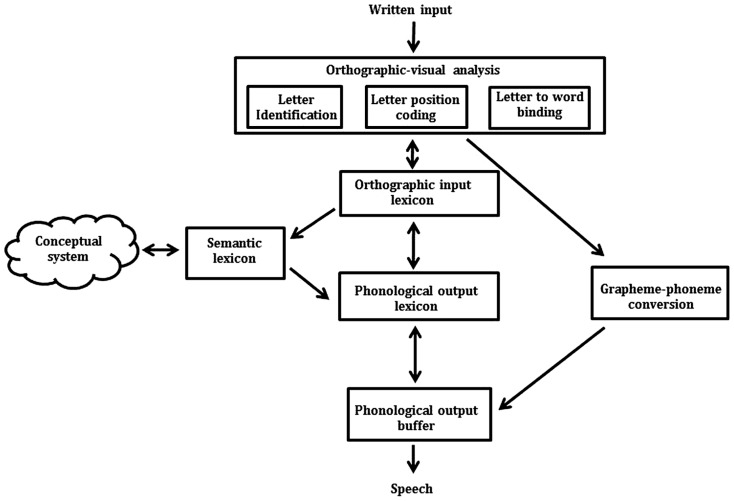
**A cognitive model of reading aloud (e.g., Friedmann and Rahamim, 2007; Kohnen et al., 2012) detailing the three reading routes: (1) the lexical route (orthographic input lexicon to phonological output lexicon), (2) the lexical-semantic route (orthographic input lexicon to phonological output lexicon via the semantic system), and (3) the non-lexical route (grapheme–phoneme conversion).** Double-headed arrows indicate feed-forward and -backward activation.

There are four studies that have investigated the locus of impairment in developmental LPD – two in Hebrew (Friedmann and Rahamim, 2007; Friedmann et al., 2010a), one in Arabic (Friedmann and Haddad-Hanna, 2012), and most recently one in English (Kohnen et al., 2012). All four studies have used the cognitive model of reading aloud illustrated in **Figure 1** to identify the locus of impairment in LPD. Following this model, when a word is encountered in print, its visual properties undergo orthographic-visual analysis. This stage involves identifying the word’s letters, coding the position of the letters within the word, and binding the letters to the word. Following these initial computations, the word is processed via three routes: (1) the lexical route (orthographic input lexicon to phonological output lexicon), (2) the lexical-semantic route (orthographic input lexicon to phonological output lexicon via the semantic system), and (3) the non-lexical route (grapheme–phoneme conversion). Typically, the lexical and lexical-semantic routes successfully process all words within a reader’s orthographic input lexicon (storage for familiar words) but fail to process non-words. In contrast, the non-lexical route successfully sounds out non-words and words that follow typical letter to sound rules (“regular words” such as *surf*, *blame*, and *hand*), but fails to provide accurate pronunciation for irregular words (such as *yacht, come*, and* friend*). According to the model, after the written input has progressed through these routes, the phonemes that make up the word are assembled and held active in the phonological output buffer until a verbal response is made.

Using this model, previous research has proposed three possible loci for the migration errors seen in LPD (Friedmann and Rahamim, 2007; Kohnen et al., 2012). First, the migration errors may occur at the phonological output buffer as the phonological code is being prepared for pronunciation. Strong evidence against this hypothesis comes from the observation that children with LPD perform within the average range on standardized tests that draw heavily on the phonological output buffer (e.g., phonological awareness and verbal short-term memory assessments; Friedmann and Rahamim, 2007; Kohnen et al., 2012). Furthermore, Kohnen et al. (2012) reported that the majority of the migration errors made by their sample of English LPDs could not be attributed to the swapping of phonemes in the output buffer. For example, the swapping of the phonemes in *cloud* (/k/ /l/ /aw/ /d/) does not create the migration error “could” (/k/ /℧/ /d/; Kohnen et al., 2012). Rather, the deficit causing this error must occur before the graphemes in the word have been converted into their appropriate phonemes.

Second, migration errors may occur due to an orthographic input lexicon deficit. On this account, LPDs are proposed to have fewer lexical entries in their orthographic input lexicon (i.e., have a smaller sight-word vocabulary) than is typical for their age. When the lexical entry matching a target word cannot be found in the lexicon, a lexical guessing strategy is adopted resulting in an error that is visually similar to the target word. This possibility is unlikely however, as LPDs have been found to read non-migratable, irregular words (e.g., *yacht*) as proficiently as their peers, indicating that their orthographic input lexicon is intact (Friedmann and Rahamim, 2007; Kohnen et al., 2012). Furthermore, if the migration errors made by LPDs are the result of lexical guessing, they should also make other lexical similarity errors, such as substitution errors (e.g., reading *slime* as “slide”). This is not the case – their reading errors appear to be selective to the transposition of letters within words (Friedmann and Rahamim, 2007; Kohnen et al., 2012).

The third and final possibility following **Figure 1** is that LPD is caused by a deficit specific to the coding of letter positions within words at the orthographic-visual analysis stage of reading. Of the three possible deficits (phonological output buffer, orthographic input lexicon, and orthographic-visual analysis), an orthographic-visual analysis deficit currently provides the most parsimonious explanation for the available data. Two pieces of evidence suggest that LPD is caused by an orthographic-visual analysis deficit. First, in Hebrew, LPDs have been found to make excessive migration errors on a same-different decision task (e.g., responding “same” to *s****l****i****m****e-s****m****i****l****e;* Friedmann and Rahamim, 2007; Friedmann et al., 2010a). Two of the three cases of English LPD reported by Kohnen et al. (2012) also showed this effect. Because the same-different decision task is thought to tap prelexical processing (see Besner et al., 1984; Kinoshita and Norris, 2009), LPDs’ poor performance on this task has been taken as evidence for an orthographic-visual analysis deficit (Kohnen et al., 2012). Second, in Hebrew, LPDs have been found to make more word responses to migratable items (e.g., reading *slime* as “smile,” and *forg* as “frog”) as well as non-word responses (e.g., reading *pilf* as “plif”), indicating that the cognitive mechanism that is defective in LPD is common to both lexical and non-lexical routes (Friedmann and Rahamim, 2007). There are two components of the model that are common to both routes: orthographic-visual analysis and the phonological output buffer. As previously outlined, there is strong evidence refuting a phonological output buffer deficit account of LPD. Therefore, the finding that LPDs in Hebrew make more word and non-word responses to migratable items has been taken as evidence for an orthographic-visual analysis deficit, which then has knock on effects to both lexical and non-lexical reading.

There are, however, two pieces of data that appear inconsistent with an orthographic-visual analysis deficit account of English LPD. First, one of the three LPD cases reported by Kohnen et al. (2012) did not make excessive migration errors on a same-different decision task. As the same-different decision task should reveal an orthographic-visual analysis deficit, this finding may suggest that the migration errors made by this case (identified as EL) are not caused by this deficit. Second, while the LPDs in Kohnen et al.’s (2012) study made more word responses to migratable items (e.g., reading *slime* as “smile,” and *forg* as “frog”) than controls, they did not make more non-word migration responses than controls (e.g., reading *pilf* as “plif”). This finding proves problematic for an orthographic-visual analysis deficit account of English LPD, as a deficit at the initial, orthographic-visual analysis stage of reading should produce migration errors in both lexical and non-lexical reading. The aim of the present study was to follow up on these two unexpected findings to clarify the locus of impairment in English LPD.

One plausible reason why EL did not make excessive migration errors on the same-different decision task is that he was adopting a strategy during the task whereby he compared each letter across the two words. In Kohnen et al.’s (2012) task, participants were presented with two words side by side, and were given as much time as they needed to make their response. As Kohnen et al. (2012) have suggested, these task conditions give participants the opportunity to compare each letter across the two words, rather than comparing the two words to one another as is intended by the task. If attention is focused on each individual letter, each letter’s position is no longer processed in relation to the position of the other letters within the word. This means that letter positions will less likely be confused, and migration errors will less likely be made.

Additionally, there are two plausible reasons why the LPDs in Kohnen et al.’s (2012) study may not have made excessive non-word migration responses, where the order of letters in a non-word stimulus is confused, resulting in a non-word response (e.g., reading *pilf* as “plif”). First, while letters in familiar words are thought to be processed in parallel via the lexical routes, letters in non-words are thought to be processed serially via the non-lexical route (Rastle and Coltheart, 1998; Friedmann and Rahamim, 2007). The serial processes that underpin non-word reading might therefore reduce the likelihood that an LPD will make non-word migration errors (Friedmann and Rahamim, 2007; Kohnen et al., 2012). Second, research in both Hebrew and English has shown that there are specific variables that influence whether or not LPDs make word migration errors. For example, LPDs are most likely to make a word migration error when a low-frequency word can migrate into a higher frequency word via the transposition of two adjacent, internal letters [e.g., reading *trail* (frequency = 18) as “trial” (frequency = 58)]. It is plausible, therefore, to hypothesize that there is also a set of variables that influence whether a non-word migration error will be made, and that variation across item sets on such variables might account for differences in results.

### THE PRESENT STUDY

The aim of this multiple single-case study with three English-speaking LPDs was to replicate and extend previous research regarding the locus of impairment in LPD.

First, we aimed to replicate previous findings suggesting that LPD is not caused by a phonological output buffer deficit. We then sought to replicate the finding that the migration errors seen in LPD are not the result of lexical guessing due to an orthographic input lexicon deficit.

Following this, we aimed to address two findings that appear to be inconsistent with an orthographic-visual analysis deficit account of LPD. The first inconsistent finding is that EL, one of Kohnen et al.’s (2012) LPDs, did not make more migration errors on a same-different decision task than controls. The second finding that appears at odds with this account is that all three LPDs in Kohnen et al.’s (2012) study did not make more non-word migration responses (e.g., reading *pilf* as “plif”) than controls. The present study therefore aimed to extend Kohnen et al.’s (2012) study by modifying the same-different decision task and the non-word reading task in an attempt to clarify the locus of impairment. Specifically, we extended Kohnen et al.’s (2012) work by (1) administering a sequential presentation variant of the same-different decision task, (2) including a consonant–string condition in the same-different decision task, and (3) manipulating the bigram frequency of the non-words presented in the reading aloud task.

A sequential variant of the same-different decision task was administered to eliminate a possible letter-by-letter matching strategy. That is, rather than presenting the words side by side, where a direct comparison between each word’s letters can be made, we presented items one after the other. Under sequential presentation, we expected all three LPDs in the present study to be significantly poorer than controls at detecting when two migratable words are different. To provide a further test of the orthographic-visual analysis deficit account of LPD, we included a consonant–string condition in the task. If LPD is due to a letter position coding deficit at the orthographic-visual analysis stage of reading, then LPDs should be poorer than controls at identifying when two migratable items are different from one another, regardless of the lexicality of the items.

In the present study, we also manipulated the bigram frequency of the non-words in the reading aloud task. One plausible reason why Kohnen et al.’s (2012) LPDs did not make more non-word migration errors than controls when reading aloud non-words (e.g., reading *pilf* as “plif”) is that there may be various factors that influence whether or not a non-word migration error will be made. Previous research has shown that the written frequency of a word’s migration counterpart, relative to the item itself, influences whether or not a migration error will be made. For example, Friedmann and Gvion (2001) found that the most common migration error made by LPDs was the reading of a non-word (which by definition has a written frequency of 0) as a word (e.g., *coisun* read as “cousin”). The next most common migration error was the reading of a word as its higher frequency counterpart [e.g., *trail* (frequency = 18) read as “trial” (frequency = 58)]. Following these findings, it is plausible to hypothesize that the bigram frequency of the non-word migration counterpart, relative to the bigram frequency of the non-word itself, will influence whether or not a non-word migration error will be made. Our exploratory hypothesis was therefore that LPDs would be more likely to migrate a low bigram frequency non-word into its higher bigram frequency non-word counterpart [e.g., reading *plif* (BF = 180) as “pilf” (BF = 1251)].

## MATERIALS AND METHODS

Ethics approval for this project was granted by Macquarie University Human Research Ethics Committee. Participants and their parents gave verbal and written consent to their involvement in the study.

### PARTICIPANTS

Participants in this study were three children: LM, EL, and LL. LM, was a 9-year 8-month-old girl in her second semester of grade 4 when we first met her and was homeschooled by her mother^1^. EL was a participant in Kohnen et al.’s (2012) study and was recruited for the present study when he was 9 years 8 months old and about to commence grade 5 at a mainstream school. Our third participant, LL, was an 11-year 9-month-old girl who had commenced grade 7 at a mainstream school two weeks before we met her.

All three children were initially referred to us because their parents were concerned about their spelling ability. Their reading skills were reported by their parents to be within the average range for their age. Both LM and LL’s hearing and vision were reported as normal. EL had long-sightedness and astigmatism, which were corrected for with glasses. He had also been diagnosed with pendular nystagmus (involuntary repetitive rhythmic movement of eyes from side to side). All three children had no diagnoses of developmental delay or difficulties [e.g., AD(H)D, SLI].

Each LPD’s performance on the standardized tests used to assess for a phonological output buffer deficit was compared to the test’s age-appropriate normative data. Each LPD’s performance on the experimental tasks was compared to a control group of average readers without LPD. We recruited two different grade-matched control groups. Six grade 4 controls were used as a control group for LM and EL (*M* age = 10 years 1 month, SD age = 2 months). Two grade 6 controls and three grade 7 controls were used as a control group for LL (*M* age = 12 years 3 months, SD age = 7 months).

### PROCEDURE

Participants were tested over multiple testing sessions at Macquarie University. Testing sessions went for between 90–150 min in length including breaks. All relevant property statistics for the experimental tasks were derived from N-Watch (Davis, 2005). All experimental reading aloud tasks and the visual lexical decision task were administered using flash cards. Unless otherwise specified, Crawford and Garthwaite’s (2002) *t*-test was used to compare each LPD’s task performance to controls, and Fisher’s exact was used to compare each LPD’s performance on one condition to another condition.

## RESULTS

### TESTS DETERMINING ELIGIBILITY

LM, EL and LL were identified as having LPD based on their scores on the Letter Position Test (LetPos: Kohnen et al., 2014). The LetPos is a reading aloud test consisting of 60 anagram words (30 anagram pairs, e.g., *slime – smile*), presented over two pages. There are three types of errors that can be made on this test: “migration errors” (reading a word as its migration partner, e.g., reading *slime* as “smile”), “word errors” (reading a word as any word other than its migration partner, e.g., reading *slime* as “slide”), and “other errors” (reading a word as a non-word, e.g., reading *slime* as “slome”). The normative data for the LetPos was collected in the final term of the school year. LPDs were selected on the basis that their LetPos performance was more than one standard deviation below the mean for “migration errors,” and within one standard deviation of the mean for “word errors” and “other errors,” when compared to the grade-appropriate normative data.

LPD participants were also selected to have no obvious reading problems, other than the reading of migratable words. Specifically, they were selected only if they had normal irregular word and non-word reading, as assessed by the Castles and Coltheart Reading Test (CC2: Castles et al., 2009). Both LM and EL were within the average range for their age (an *z*-score between –1 and +1) on both the irregular word and non-word reading components of the test. While LL was within the average range on the irregular word component of the CC2, she was below average on the non-word reading component of the test^2^. She was included in the study, however, because her non-word reading errors appeared to stem from an underlying problem with reading letters in their correct order. For example, LL made non-word migration errors such as reading *borp* as “brop.” When these migration errors were removed from her score, her non-word reading was within the average range.

Control participants were selected to be average on the irregular word and non-word reading subtests of the CC2 and to be within one standard deviation of the mean on each component (migration, word and other errors) of the LetPos.

### ASSESSING THE PHONOLOGICAL OUTPUT BUFFER

A phonological output deficit should manifest itself in poor performance on tasks that require phoneme production and/or manipulation. To investigate whether LPD is caused by a phonological output buffer deficit, LM, EL and LL were assessed on phonological awareness, speed of lexical retrieval and verbal short-term and working memory. If their migration errors are caused by a phonological output buffer deficit, they should be below average on these tasks compared to age-appropriate normative data.

Phonological awareness was assessed using the Segmenting Non-words and Phoneme Reversals subtests of the Comprehensive Test of Phonological Processing (CTOPP, Wagner et al., 1999). In the Segmenting Non-words subtest children are given a series of non-words, which they are asked to repeat, and then say one sound at a time (e.g., “dray, d – r – ay”). In the Phoneme Reversal subtest children are asked to first repeat a non-word, and then to reverse the sounds to make it sound like a real word (e.g., “nus, sun”).

Speed of lexical retrieval was assessed using the Rapid Naming subtests of the CTOPP. LPDs were assessed on their ability to rapidly name letters, digits, objects and colors, which were each assessed separately. In these subtests, LPDs were asked to name 36 items presented on a single page as quickly as they could.

The Repetition of Nonsense Words subtest of the NEPSY (Korkman et al., 1998) and the Digit Span subtest of the Weschsler Intelligence Scale for Children Fourth Edition (WISC-IV; Wechsler, 2003) were used to assess verbal short-term and working memory. In the Repetition of Nonsense Words subtest, children are asked to repeat non-words (e.g., *bu-lεks-tıs*). The Digit Span subtest has two components – Forward Digit Span, and Backwards Digit Span. In the Forward Digit Span children are asked to repeat strings of digits in the same order as they heard them, and in the Backwards Digit Span subtest children have to repeat strings of digits in reverse order.

**Table 1** shows that all LPD participants were within (or even above) the average range (*z*-score between -1 and +1) on all nine measures of phonological processing. In addition LM, EL, and LL were asked to orally repeat the words after the experimenter for which they had previously made a migration error on in a reading aloud task. Each LPD performed this task without making a single migration error.

**Table 1:** *Z* scores on standardized tests used to assess for a phonological output deficit (average range is between –1 and +1).

		LM	EL	LL
Phonological awareness	Segmenting nonwords (CTOPP)	0.33	1.67	1.67
	Phoneme reversals (CTOPP)	0.33	0.67	–0.67
Lexical retrieval	Rapid naming (CTOPP)		
	Digits	1.33	0.33	–0.67
	Letters	1.00	0.67	–1.00
	Colors	1.00	0.33	–1.00
	Objects	1.33	–0.67	–0.33
Verbal memory	Digit span (WISC-IV)				
	Forward	1.00	0.33	1.00
	Backward	0.00	–0.33	0.67
	Repetition of nonsense words (NEPSY)		1.00	1.00	0.33

Taken together, these findings indicate that the migration errors made by the three LPDs in the present study cannot be attributed to a phonological output buffer deficit.

### ASSESSING THE ORTHOGRAPHIC INPUT LEXICON

To investigate whether LM, EL and LL have an orthographic input lexicon deficit we administered a reading aloud non-migratable, irregular words task. Irregular words were used to ensure that access to the orthographic input lexicon was obligatory for a correct response to be made. If LPDs have an orthographic input lexicon deficit, they should be poorer at this task than controls.

To explicitly test whether their excessive migration errors are the result of lexical guessing, we administered two tasks: a reading aloud migratable and substitution words task, and a visual lexical decision task. If LPDs’ migration errors are the result of lexical guessing, they should make more substitution errors than controls on a reading aloud task (e.g., reading *track* as “trick”), as well as more substitution errors on a visual lexical decision task (e.g., accepting *esho* (derived from *echo*) as a word).

#### Reading non-migratable, irregular words

Participants were asked to read aloud 87 non-migratable words which were selected to contain at least one letter-sound rule that was atypical (e.g., pearl, cousin) according to Regcelex (Baayen et al., 1995), a program used to compute the rule based pronunciation of a letter-string (Coltheart et al., 2001). Because we were interested in each LPD’s lexical reading skills, errors that appeared to stem from a difficulty in ordering letters in words (e.g., reading *chalk* as “chlak”) were removed from the error analysis. Both LM and EL made 12.64% errors on this task, which was not significantly different from their control group, who made 9.58% errors (SD = 2.48%; *t* = 1.14, *p* = 0.15 one-tailed). LL made 6.90% errors on this task, which was not significantly different from her control group who made 8.28% errors (SD = 2.36%; *t* = 0.53, *p* = 0.31 one-tailed).

Eighteen of the 87 experimental words were items that had already been administered in the irregular word reading component of the CC2. We therefore conducted an additional analysis including irregular words that were not part of the CC2 (*N* = 69). All three LPD’s made as many errors as controls in this additional analysis (all* p* > 0.15 one-tailed).

This finding suggests that LM, EL and LL have as many entries in their orthographic input lexicon as controls, and that they have no difficulty in accessing these entries.

#### Reading aloud migratable and substitution words

Participants read aloud 58 migratable words, which were created from 29 word pairs that were different via the transposition of two internal letters (e.g., *slime-smile*). Migratable words were intermixed with 30 substitution words created from 15 pairs of words that were different via the substitution of a single internal letter (e.g., *track-trick*). Substitution words were matched as closely as possible to migratable words on length (migratable: *M =* 5.07, SD = 0.53; substitution: *M* = 5.07, SD = 0.69), relative written frequency between a word and its partner (migratable: *M =* 27.51, SD = 36.83; substitution: *M* = 36.61, SD = 36.62), and the number of substitution neighbors (migration: *M =* 4.86, SD = 3.48; substitution: *M* = 4.90, SD = 3.18). The item pairs were presented over separate tasks such that participants did not read a word and its partner in the same task. These words were intermixed with 122 words, which were not used to address the research questions in the present study.

Three error types were analyzed: (1) migration errors, where a migratable word was read as its partner, (2) substitution errors, where a substitution word was read as its partner, and (3) “*N*” errors, which included substitution errors (e.g., reading *slime* as “slide”), addition errors (reading *slime* as “slimes”), and deletion errors (reading *slime* as “slim”) made on all migratable and substitution words. Incorrect reading responses that were potentially due to sounding the word out rather than one of these three error types (e.g., reading *bread* as “breed”) were not included in the analysis.

The results are outlined in **Table 2**. All three LPDs made more migration errors than controls (LM: *t* = 21.95, *p* < 0.001 one-tailed; EL: *t* = 9.49; *p* < 0.001 one-tailed; LL: *t* = 4.81, *p* < 0.01 one-tailed). Because there was no variance in the number of substitution errors made by controls, a Fisher’s exact test was used (instead of Crawford’s *t*-tests) to compare LPDs’ performance to their respective control groups. All three LPDs made as many substitution errors as controls (all *p* > 0.5 one-tailed). Both LM and EL made as many *N* errors as controls (both *t* = 1.11, *p* = 0.16 one-tailed). Because there was no variance in the number of *N* errors made by LL’s control group, a Fisher’s exact test was used instead of Crawford’s *t*-test, which indicated that she made as many N errors on the task as controls (*z* = 0.71, *p* = 0.24 one-tailed).

**Table 2:** Percentage of errors on reading aloud words in the migration and substitution conditions.

	LM	EL	LM and EL controls	LL	LL controls
Migration errors	27.59^***^	15.52^***^	6.32 (0.89)	12.07^**^	2.07 (1.89)
Substitution errors	3.33	0.00	0.00 (0.00)	0.00	0.00 (0.00)
*N* errors	2.27	2.27	0.95 (1.12)	2.27	0.00 (0.00)

*Numbers in parentheses denote standard deviation of the mean for control groups.*

** *p* < 0.01, ***p < 0.001 compared to control group.

The finding that LM, EL and LL’s reading errors were selective to the migration of letters within words suggests that their LPD cannot be attributed to lexical guessing.

#### Visual lexical decision

A visual lexical decision task was also administered to determine whether migration errors made by LPDs were the result of lexical guessing. Forty non-migratable words formed the word condition in this task. Three non-word conditions were created by modifying the word items – a migratable non-word condition (*coisun* (derived from *cousin*); *N* = 16), a single-substitution non-word condition (*eamly* (derived from *early*), *N* = 12), and a double-substitution non-word condition (*provare* (derived from *private*); *N* = 12). Single and double substitution items were included because both have previously been used in research as a comparison condition for migratable items (e.g. Perea and Lupker, 2004; Perea and Fraga, 2006; Beyersmann et al., 2011, 2012, 2013).

Items in the migratable non-word condition were matched as closely as possible to items in the single- and double-substitution condition on bigram frequency (migration condition: *M* = 719.04, SD* =* 415.91; single-substitution condition: *M* = 578.54, SD = 336.08; double-substitution condition: *M* = 713.68, SD = 553.36), and the written frequency of the words that they were derived from (migratable *M* = 87.56, SD* =* 125.20; single-substitution: *M* = 96.89, SD = 116.29; double-substitution: *M* = 72.64, SD = 112.39). Words and non-words were intermixed with 112 additional items, which were not used to address the research questions in the present study. Items were presented over two separate tasks, such that a non-word and the word it was derived from were not presented in the same task.

So that we could be relatively certain that a “word” response to a non-word was due to the participant misreading the non-word as the word it was derived from, non-words in the migration condition and the double-substitution condition did not have a single substitution neighbor. Furthermore, the non-words in the single substitution condition did not have a single substitution neighbor other than the word that they were derived from. To further ensure that participants’ “word” responses were due to their misreading of the non-word as its word partner, we removed non-words derived from words that participants did not know. We determined whether or not a participant knew a word based on their performance on the “word” condition of the visual lexical decision task, and their reading aloud of these words. If a participant could not read aloud the word *and* did not recognize the word in the visual lexical decision task, the word was defined as unknown, and hence its non-word counterpart was removed from their individual analysis. This comprised 5.00% of LM’s data, 2.50% of EL’s data, and 2.92% (SD = 3.68%) of their control group’s data. For LL, 2.50% of her data was removed, and 1.00% (SD = 2.24%) of her control group’s data was removed.

The results are outlined in **Table 3**. All three LPDs accepted more migratable non-words as words than controls (LM: *t* = 3.59, *p* = 0.01 one-tailed; EL: *t* = 2.59, *p* = 0.02 one-tailed; LL: *t* = 2.90, *p* = 0.02 one-tailed). Both EL and LL accepted as many single and double substitution non-words as words as controls (both *t* < 1.12, *p* > 0.16 one-tailed). LM, however, accepted more single and double substitution non-words as words than controls (single: *t* = 2.51, *p* = 0.03 one-tailed; double: *t* = 4.54, *p* = 0.003 one-tailed).

**Table 3:** Percentage of migration errors, single-substitution (sub) errors and double-substitution (sub) errors on the visual lexical decision task.

	LM	EL	LM and EL controls	LL	LL controls
Migration errors	64.29^*^	53.33^*^	24.89 (10.16)	43.75^*^	12.58 (9.82)
Single-sub errors	33.33^*^	8.33	6.94 (9.74)	8.33	3.33 (4.56)
Double-sub errors	25.00^**^	8.33	2.90 (4.51)	0.00	3.33 (7.45)

*Numbers in parentheses denote standard deviation of the mean for control groups.*

* *p* < 0.05, **p < 0.01 compared to control group.

The finding that EL and LL’s excessive errors on the visual lexical decision task were selective to the migration condition suggests that their migration errors are not the result of lexical guessing. In contrast, LM’s excessive errors on the task were not selective to the migration condition – she also made more substitution errors on the task than controls. This finding suggests that a lexical guessing strategy may have been the cause of LM’s migration errors on the visual lexical decision task.

### ASSESSING THE ORTHOGRAPHIC-VISUAL ANALYSIS STAGE OF READING

To investigate whether LPD is caused by an orthographic-visual analysis deficit, we administered a sequential same-different decision task and a reading aloud non-words task. If LPD is caused by an orthographic-visual analysis deficit, LPDs should make more migration errors than controls on tasks that tap prelexical processing (e.g., same-different decision) since orthographic-visual analysis is a prelexical process. Furthermore, if their migration errors are caused by an orthographic-visual analysis deficit, LPDs should make more migration errors than controls during lexical and non-lexical reading.

#### Sequential same-different decision

The sequential same-different decision task consisted of 139 word pairs and 139 consonant–string pairs^3^, which were four or five letters in length. Half of the items were the same (e.g., *beard–beard*; *bfgsk–bfgsk*), and half were different (*beard–bread*; *bfgsk–bfsgk*). Half of the items in the different condition were different via the transposition of internal letters (e.g., *trial–trail*), and half were different via the substitution of a single letter (e.g., *chuck–check*). Items were included in both the same and the different condition (i.e., participants made responses to both *trial–trail* and *trial–trial*). Six versions of the task were created and presented over two sessions, such that participants only saw one version of the item (either in the same *or* in the different condition) in a single session. These 280 items were intermixed with an additional 280 items (half same, half different), which were not used to address the research questions in the present study.

**FIGURE 2:**
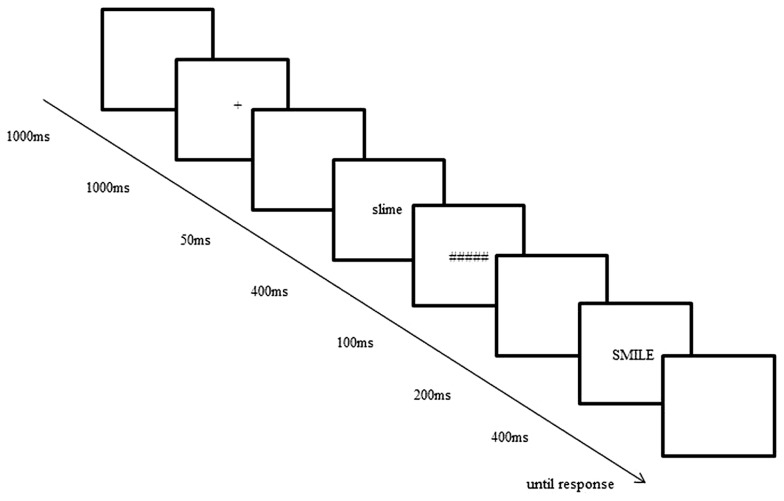
**Schematic of a single same-different decision trial**.

Same-different decision trials were presented using DMDX software (Forster and Forster, 2003). A schematic of a single trial is outlined in **Figure 2**. The first item was both backwards masked and presented in a different case to the second item to ensure that participants could not match the items based on low-level perceptual overlap. Participants were instructed to press a button with their right hand if they thought the two items were the same, and to press a button with their left hand if they thought the two items were different. Participants were given eight practice trials before commencing the task. No performance-based feedback was given to participants at any stage during the task.

As LPDs have been found to have intact letter identification skills (Friedmann and Rahamim, 2007; Kohnen et al., 2012), the substitution condition was used as an indication of baseline performance on the task. If LPD is due to an orthographic-visual analysis deficit, LPDs should be poorer than controls at detecting a difference between two migratable items (e.g., *slime–smile*), relative to the baseline condition (e.g., *tiger–timer*).

**Table 4** displays participants’ accuracy on the different conditions (i.e., their ability to detect that two items are different). Participants’ *d*′ scores based on their hits (correctly responding “different” to two different items e.g., *slime–smile*) and false alarms (incorrectly responding “different” to two same items e.g., *slime–slime*) on the migration and substitution condition are also included in **Table 4**.

**Table 4:** Percentage accuracy for the different migration (mig) and substitution (sub) conditions on the same-different decision task, and *d*′ scores.

		LM		EL		LM and EL controls		LL		LL controls	
		Accuracy	*d*′	Accuracy	*d*′	Accuracy	*d*′	Accuracy	*d*′	Accuracy	*d*′
Words	Mig	40.00	1.22	54.29	1.00	89.52 (6.68)	3.03 (0.68)	71.43	1.83	94.29 (5.35)	3.69 (0.59)
	Sub	80.00	3.01	100.00	3.35	93.81 (2.81)	3.25 (0.45)	91.43	2.60	96.00 (8.94)	3.85 (0.99)
Consonants	Mig	41.18	1.08	67.65	0.34	65.69 (8.86)	0.73 (0.53)	85.29	0.61	74.71 (25.01)	1.66 (1.08)
	Sub	22.86	0.43	80.00	0.78	60.00 (15.65)	1.16 (0.45)	65.71	0.29	73.71 (15.31)	1.68 (0.78)
Non-words	Mig****	58.33****	1.67****	54.17****	0.96****	88.54 (9.85)	3.24 (0.77)****	66.67****	1.35****	88.33 (11.56)****	2.83 (0.99)
	Sub	70.83	1.99	100.00	2.92	96.88 (6.25)	3.85 (0.78)	87.50	2.02	95.83 (5.10)	3.22 (0.75)

*Numbers in parentheses denote standard deviation of the mean for control groups.*

All statistical analyses for the task were based on participants’ accuracy on the different migration condition relative to their accuracy on the different substitution condition, using the Revised Standardized Difference Test (RSDT: Crawford and Garthwaite, 2005). All three LPDs were significantly poorer than controls at detecting that two migratable words were different relative to the substitution condition, however this only reached significance for EL and LL (EL: *t* = 4.68, *p* = 0.003 one-tailed; LL: *t* *=* 2.82, *p* = 0.02 one-tailed; LM: *t* = 1.74, *p =* 0.07). All three LPDs were not significantly poorer than controls at detecting that two migratable consonant strings were different, relative to the substitution condition (all *t* < 1.10, *p* > 0.16).

The finding that all three LPDs were no poorer than controls at detecting a difference between two migratable consonant–strings seems inconsistent with an orthographic-visual analysis deficit account of LPD. If LPD is caused by an orthographic-visual analysis deficit, then LM, EL and LL should be poorer than controls at detecting a difference between two migratable items, regardless of their lexicality.

However, this result may have been due to participants not having enough time to process the entire consonant–string. Letters in words are thought to be processed in parallel as a single unit of information. In contrast, there is no higher-order representation for consonant strings, and therefore each letter needs to be processed serially as its own unit of information. The limited stimulus presentation time in the task (400 ms) may have therefore meant that children only had enough time to process the beginning letters of the items in the consonant–string condition. If only the beginning letters are processed, then a correct response to many of the items in the different migration condition would require intact letter identification skills, but not necessarily intact letter position coding skills. For example, if participants are presented with the consonant–string pair *s****t****l****k****d-s****k****l****t****d*, but they only have enough time to process the first three letters of the consonant string, *s****t****l-s****k****l*, participants need only detect that the letter identities *t* and *k* are different from one another to make a correct response. If participants were only processing the beginning letters of the consonant–string pairs, then the finding that LPDs did not make more errors on the migration condition is not surprising, as LPDs have been found to have intact letter identification abilities (Friedmann and Rahamim, 2007; Kohnen et al., 2012).

One way to investigate whether or not participants had enough time to process all letters in the consonant–string condition is to see whether there is a position effect. If participants did not have enough time to process the entire consonant–string, we should find that they are better at detecting a difference between two consonant strings if the letters are different at the beginning of the pair, than if the letters are different at the end of the pair.

In a *post hoc* analysis, we explored whether there was merit in this alternative hypothesis. Items that differed via the substitution of a single letter in the first internal position of the word (e.g., *n****k****dcg-n****j****dcg*) were classified as having a “beginning difference,” and items that differed via the substitution of a single letter in the final internal position of the word (e.g., *fkm****z****d-fkm****t****d*) were classified as having an “end difference.” The substitution condition rather than the migration condition was used because many of the different migratable items had both a beginning and end difference (e.g., *x****t****k****j****d-x****j****k****t****d*).

All participants were combined to form one group for this item analysis. We used a Wilcoxon matched pairs test to compare the proportion correct on the two groups of items. Participants identified significantly more beginning differences (74.60%) in the consonant–string condition than end differences (58.574%;* z =* 2.51, *p* = 0.006 one-tailed). In contrast, participants identified as many beginning differences (93.57%) in the word condition as end differences (94.76%; *z =* 0.51, *p* = 0.304 one-tailed).

Following this finding, we decided to administer a same-different decision task with orthographically legal non-words (e.g., *scirm-scrim*). While the letters in legal non-words are not thought to be processed in parallel like words, the letters can be mapped onto a higher-order representation. For example, the consecutive letters *i* and *r* in the non-word *scirm* can be mapped onto the digraph *ir*. That is, the letters in legal non-words can be “chunked” (*s*, *c*, *ir* and *m*) and, for this reason, are likely to be processed faster than consonant–strings which cannot be chunked.

The non-word same-different decision task consisted of 96 non-word pairs. Forty-eight of the pairs were in the same condition, and 48 were in the different condition. Half of the items in the different condition were different via the transposition of two internal letters (e.g., *scirm-scrim*), and half were different via the substitution of a single letter (e.g., *froy-floy*). The same condition consisted of 48 non-word pairs. In contrast to the word and consonant string same-different decision task, non-words in the same condition were a new set of items, not derived from the items in the different condition (i.e., participants did not see *scirm-scrim* and *scirm-scirm*). Non-words were presented to participants during a single task, and under the same presentation conditions as described for the words and consonant–strings task.

By the time we assessed LM and EL on this task they were in the second semester of grade 5. Therefore, we compared their performance on this task to a new control group of 4 children in their second semester of grade 5.

**Table 4** displays participants’ accuracy on the different conditions. Participants’ *d*′ scores based on their hits (correctly responding “different” to two different items e.g., *scirm-scirm*) and false alarms (incorrectly responding “different” to two same items e.g., *garp-garp*) on each condition are also included in **Table 4**. False alarms were calculated from participants’ performance on all 48 items in the same condition.

EL was significantly poorer than controls at detecting when two migratable non-words were different relative to the substitution condition (EL: *t =* 4.47, *p* = 0.01 one-tailed). LM and LL, however, did not show this effect (both *t* < 1.64, *p >* 0.10). We assessed for a position effect in the same way as we did for the consonant–string and word items. Participants correctly identified as many beginning differences (95.14%) as end differences (91.67%; *z =* 0.54, *p* = 0.30 one-tailed), indicating that they had enough time to process the entire letter string.

The finding that all three LPDs made more word migration errors than controls on a sequential same-different decision task is consistent with an orthographic-visual analysis deficit account of LPD, as is the finding that EL made more non-word migration errors on the task. The finding that LM and LL did not make more non-word migration errors on the sequential same-different decision task is, however, inconsistent with an orthographic-visual analysis deficit and will be followed up in the discussion.

***Reading aloud non-words.*** Non-words were created from 25 non-word pairs which were migratable via the transposition of two internal adjacent letters (e.g., *torm–trom*). Pairs were selected to have a significant difference in bigram frequency between the two non-words (lower bigram frequency counterpart: *M* = 789.56 SD* =* 594.36; higher bigram frequency counterpart: *M* = 1389.80, SD* =* 841.41). Non-words were selected to match their migration partner as closely as possible on substitution *N* (lower bigram frequency counterpart: *M* = 2.44, SD = 2.38; higher bigram frequency counterpart: *M* = 3.00, SD = 2.65). Non-words were randomized and intermixed with 25 additional monosyllabic non-words that were not used to answer the research questions in this paper. Three versions of the task were created such that participants did not see a non-word and its migration partner in the same task. Participants were told that all items were nonwords before commencing the task.

The results from the nonword reading task are presented in **Table 5**. Both LM and LL made significantly more nonword migration errors on the task than controls (LM: *t* = 6.46, *p* < 0.001 one-tailed; LL: *t* = 2.96, *p* = 0.02 one-tailed) and made as many non-migration related errors as controls (LM: *t* = 0.04, *p* = 0.48 one-tailed; LL: *t* = 1.82, *p* = 0.07 one-tailed). EL did not make more nonword migration errors than controls (*t* = 0.18, *p* = 0.43 one-tailed) and made more non-migration related errors than controls (*t* = 2.95, *p* = 0.02 one-tailed).

**Table 5:** Percentage of migration errors (mig error) and non-migration related errors (non-mig error) on reading aloud non-words.

	LM	EL	LM and EL controls	LL	LL controls
Mig error	40^***^	6	5.00 (5.02)	16^*^	4.40 (3.58)
Non-mig error	12	36^*^	12.33 (7.42)	20	7.20 (6.42)

*Numbers in parentheses denote standard deviation of the mean for control groups.*

* *p* < 0.05, ***p < 0.001 compared to control group.

Following the finding that EL showed the opposite effect to that displayed by LM and LL (i.e., as many migration errors as controls, but more non-migration related errors than controls), we decided to inspect EL’s non-word reading data more closely. We found that 23% of ELs non-migration errors were what we have termed, “over-sequential” errors. An “over-sequential” error was defined as an error that appeared likely to have occurred as a result of sounding out each letter in the non-word in isolation, and then blending these sounds together to form a spoken response. For example, EL read *kerm* as /k /ε/ /r/ /m/. That is, instead of reading the letters *e* and *r* together to correctly form the sound /ər/, he sounded out these two letters separately. For two of these errors, EL first misread the non-word as its migration partner, and then self-corrected with an over-sequential error. Furthermore, for all but one of EL’s over-sequential errors, EL demonstrated that he knew the sound associated with the multi-letter grapheme he over-sequentialized by correctly producing it on at least two other items within the list. EL’s control group did not make a single “over-sequential” error on this task. This finding suggests that EL’s limited migration errors on this task (compared to the other LPDs in the study) may have been the result of him sounding out each letter in isolation of the other letters within the word.

The findings from the reading aloud non-words task suggest that LPD is most likely caused by an orthographic-visual analysis deficit. However, there appears to be variation in task performance among the three LPDs in the present study.

***Item variables influencing non-word migration errors.*** In the present study, we also explored the possibility that there may be specific item variables that influence whether or not LPDs will make non-word migration errors. Specifically, we explored whether the bigram frequency of the non-word migration counterpart relative to the bigram frequency of the non-word itself, influenced whether or not a non-word migration error will be made.

We investigated the influence of bigram frequency on non-word reading by analyzing the migration errors made by LM and LL. Specifically, we compared the number of migration errors made on the lower bigram frequency partner (*N* = 25) to the number of migration errors made on the higher bigram frequency partner (*N* = 25). The other participants’ results (EL and both control groups) were not investigated in this additional analysis as they made very few migration errors on the task. Both LM and LL read as many non-words as their higher bigram frequency migration partner (LM: 40%, LL: 8%) as they did non-words as their lower bigram frequency partner (LM: 40%; LL: 24%; both Fisher’s exact *p* > 0.12 one-tailed).

While bigram frequency was not found to mediate migration errors on this task, a *post hoc* analysis revealed that LM and LL’s migration errors were influenced by the complexity of the graphemes that made up each non-word. LM and LL were more likely to migrate a two-letter grapheme into two single-letter graphemes (e.g., reading *k****er****m* as “k**re**m”) than to migrate two single-letter graphemes into a two-letter grapheme (e.g., reading *k****re****m* as “k**er**m”). Both LM and LL were found to migrate significantly more two-letter graphemes into single letter graphemes (LM: 66.67%, LL: 33.33%) than two single-letter graphemes into a two letter grapheme (LM: 11.11%, LL: 0%, both Fisher’s exact *p* < 0.02 two-tailed).

An examination of the order of item presentation was conducted to investigate whether the errors where a two-letter grapheme migrated into two single-letter graphemes were due to participants being primed by the two single-letter graphemes. That is, we examined whether participants saw the two single letter graphemes (e.g., *f****re****mpt*) prior to making an error where they migrated a two-letter grapheme into these two-single letters (e.g., reading *k****er****m* as *k****re****m*). Of the 18 errors made by LM and LL where a two-letter grapheme was migrated into two single letters (e.g., where *k****er****m* was read as “k**re**m”), only three errors were made directly after having seen a non-word that comprised the same two single letters (e.g., *f****re****mpt*).

## DISCUSSION

This study investigated the locus of impairment in three English-speaking children with developmental LPD. Previous research has used a cognitive model of reading aloud to identify three alternative processing components that may be the cause of LPD: the phonological output buffer, the orthographic input lexicon, and orthographic-visual analysis. First, we aimed to replicate previous findings that have ruled out a phonological output buffer deficit and an orthographic input lexicon deficit account of LPD. We then went on to extend previous findings that suggest LPD is caused by an orthographic-visual analysis deficit.

### ASSESSING THE PHONOLOGICAL OUTPUT BUFFER

It is plausible to assume that the excessive migration errors made by LPDs are due to the phonemes in the phonological output buffer being swapped around before the word is pronounced. Together with previous studies, our findings strongly refute this hypothesis (Friedmann and Rahamim, 2007; Kohnen et al., 2012; see also Collis et al., 2013). All three LPDs in the present study were either within or above the average range on various standardized tests that draw heavily on a functioning phonological output buffer to be completed successfully. Furthermore, LPDs were asked to repeat a subset of the migratable words that they had previously made a migration error on in a reading aloud task. Each LPD performed this task without making a single migration error, indicating that their reading aloud errors were not caused by an inability to produce the word’s phonemes in the correct order.

In recent years, various researchers have suggested that underlying dyslexia is a phonological processing deficit (Stanovich, 1988; Snowling, 1998, 2001; Ramus, 2003). The findings from the present study indicate that, while some children with reading difficulties have phonological processing difficulties, other children’s reading difficulties are likely to reflect an alternative processing deficit. For example, surface dyslexia is most likely caused by an orthographic processing deficit (e.g., Castles and Coltheart, 1993, 1996; Broom and Doctor, 1995; Temple, 1997), attentional dyslexia is most likely caused by a letter-to-word binding deficit (Rayner et al., 1989; Friedmann et al., 2010b), and LPD is most likely caused by a letter position coding deficit (for more discussion of heterogeneity within developmental dyslexia, see Castles et al., 2010; Zoccolotti and Friedmann, 2010; McArthur et al., 2013).

### ASSESSING THE ORTHOGRAPHIC INPUT LEXICON

It is also plausible to assume that the migration errors made by LM, EL and LL are the result of lexical guessing due to an impoverished orthographic input lexicon. The finding that all three LPDs read aloud non-migratable irregular words as well as controls indicates that this is not the case. Furthermore, EL and LL made more migration errors than controls during a reading aloud task and a visual lexical decision task but did not make more substitution and *N* errors than controls. These findings indicate that EL and LL’s errors on these tasks were specific to the migration of letters within the word and were therefore not due to lexical guessing.

In contrast to EL and LL, LM made more migration errors than controls on the visual lexical decision task *and* more substitution errors on the task. This finding suggests that perhaps LM’s tendency to make excessive migration errors is the result of lexical guessing. While this finding does not fall in line with our predictions, we believe that LM’s lexical guessing was confined to this task, and that her broader tendency to make more migration errors than her peers cannot be attributed to a lexical guessing strategy. If LM’s excessive migration errors are the result of lexical guessing, then she should have been found to make more errors that are visually similar to the target word when reading aloud (e.g., reading *slime* as “slide” or “slim”) than controls. This was not the case. Like EL and LL, LM made more migration errors than controls when reading aloud, but the same amount of substitution and *N* errors.

### ASSESSING THE ORTHOGRAPHIC-VISUAL ANALYSIS STAGE OF READING

The first aim of the present study was to replicate the finding that LPD cannot be attributed to a phonological output buffer deficit or an orthographic input lexicon deficit. Our findings converge with previous research that has ruled out these two possible loci as the source of migration errors seen in LPD (Friedmann and Rahamim, 2007; Kohnen et al., 2012). Having addressed our first aim, we now turn to a discussion of our second aim: to extend the investigation of a possible orthographic-visual analysis deficit account of LPD.

The present study extended Kohnen et al.’s (2012) study in three ways: (1) administering a sequential same-different decision task, (2) administering consonant–strings and orthographically legal non-words in the sequential same-different decision task, and (3) manipulating bigram frequency in a non-word reading task. We hoped that making these changes would provide us with tasks that were more sensitive to an orthographic-visual analysis deficit, and hence enable us to draw stronger conclusions regarding the locus of impairment in English LPD.

In the present study, we administered a sequential same-different decision task to ensure that participants would be unable to adopt a strategy whereby they compare each letter in the pair to one another. We found that EL and LL made significantly more word migration errors on the task than controls. LM also showed this trend, however it did not reach significance. One key difference between the present study and Kohnen et al.’s (2012) study was EL’s performance on the same-different decision task. While EL did not make more migration errors on Kohnen et al.’s (2012) simultaneous same-different decision task, he made significantly more migration errors on a sequential variant of the task in the present study. One interpretation of this finding is that EL was adopting a letter-by-letter matching strategy during Kohnen et al.’s (2012) simultaneous same-different decision task. When he was unable to adopt this strategy during the present study, due to the sequential presentation of words, he made significantly more migration errors than controls.

An alternative interpretation of EL’s excessive migration errors on the same-different matching task in the present study is that a sequential variant of the task encourages participants to convert the word into a phonological form due to the limited presentation time of the items. It might therefore be that EL made excessive migration errors on the sequential task because he compared the words in each pair based on phonological form, whereas in Kohnen et al.’s (2012) simultaneous task, words were compared based on their orthographic form. We believe this alternative hypothesis to be unlikely for two reasons. Firstly, a wealth of research has shown that responses made on a same-different decision task are based on prelexical orthographic representations rather than phonological representations (e.g., Besner et al., 1984; Kinoshita and Norris, 2009). Secondly, EL was found to be within (or above) the average range on tests that assess phonological processing. It is therefore highly unlikely that EL’s excessive migration errors on the sequential same-different decision task could be reflecting a difficulty in comparing phonological forms.

A consonant–string condition in the same-different decision task was included in the present study under the assumption that a letter position coding deficit should manifest itself in responses to all letter-strings, regardless of lexicality. Contrary to our prediction, LPDs did not make more migration errors than controls on the consonant–string condition. We believe that this finding was due to the different mechanisms underlying the processing of letters in words and in consonant–strings. While letters in words are thought to be processed in parallel as a single unit, each letter in a consonant–string needs to be processed serially as a single unit. This means that letters in consonant–strings are likely to take longer to process than letters in words. The *post hoc* finding that participants were significantly better at identifying a difference between two consonant strings when the difference occurred toward the beginning of the consonant pair (*f****k****tzm-f****l****tzm*) than when the difference occurred toward the end of the consonant pair (*fkt****z****m-fkt****l****m*) suggests that 400ms was not enough time for participants to process the entire consonant–string. For this reason, we believe that participants’ performance on the consonant–string condition cannot be taken as evidence for or against an orthographic-visual analysis deficit account of LPD.

Following this finding, we conducted a sequential same-different decision task with orthographically legal non-words. We found that while EL made significantly more migration errors than controls on this task, LM and LL did not. The finding that EL made more word *and* non-word migration errors on a same-different decision task strongly suggests that EL’s excessive migration errors are caused by an orthographic-visual analysis deficit. In contrast, the finding that LM and LL made more migration errors on the word condition, but not on the non-word condition is not predicted by an orthographic-visual analysis deficit account of LPD. Rather, LM and LL should have been found to make more migration errors on a sequential same-different decision task, regardless of the lexicality of the items. However, LM and LL’s data are still most consistent overall with an orthographic-visual analysis deficit. Further investigations may need to focus on the interaction between lexicality effects and orthographic-visual analysis deficits in LPD.

We also administered a non-word reading task in the present study. If LPD is caused by an orthographic-visual analysis deficit, we should find that LPDs not only make more word migration errors (e.g., reading *slime* as “smile”) than controls, but also more non-word migration errors (e.g., reading *pilf* as “plif”), as a deficit at the orthographic-visual analysis stage of reading should impede both lexical and non-lexical reading. In the present study, we found that LM and LL made more non-word migration errors (e.g., reading* pilf* as “plif”) than controls. This finding is in contrast with Kohnen et al.’s (2012) finding that all three LPDs made as many non-word migration errors as controls. Interestingly, the one LPD in the present study who did not make excessive non-word migration errors (EL) was one of the three LPDs in Kohnen et al.’s (2012) study who did not make excesive non-word migration errors when reading aloud. This finding is consistent with research in Hebrew that has found that while some LPDs make non-word migration errors, others do not (Friedmann and Rahamim, 2007). EL’s over-sequential errors (where each letter was sounded out in isolation and then blended together to form a response) in the present study suggest that individual differences in strategy use might be one predictor of whether or not LPDs will make non-word migration errors.

Contrary to our exploratory hypothesis, we found non-word bigram frequency to have no influence over whether or not a migration error was made. That is, LM and LL were no more likely to read a non-word as its higher bigram frequency partner than they were to read a non-word as its lower bigram frequency partner. The majority of migration errors made by LPDs occur when two adjacent letters in the middle of a word can migrate to form a new word. Considering it is the internal letters of the non-word that are most prone to migration, it is perhaps not surprising that the bigram frequency of the entire letter-string (external letters included) did not influence whether or not a migration error occurred. Instead, it may be other factors specific to the letters that are most susceptible to migration that influence whether or not a migration error will be made.

This suggestion was supported by the *post hoc* finding that the complexity of the non-word’s internal grapheme/s influenced whether or not a migration error was made. We found that LM and LL were more likely to swap the letters in a two-letter grapheme around to form two single-letter graphemes, than to swap two single letters around to form a two-letter grapheme (i.e., *k****er****m* was read as “k**re**m” more than* k****re****m* read as “k**er**m”)^4^. One plausible explanation for this finding is that children are likely to be introduced to the sounds that the letters of the alphabet make (single-letter graphemes) before they are introduced to the sounds that two letters of the alphabet make together (two-letter graphemes). What this finding might therefore reflect is an age of acquisition effect. When the non-lexical route is provided with ambiguous letter position information, the default may be to resort to the letter-sounds that were first learnt. Future studies may seek to further our *post hoc* finding by directly testing the hypothesis that some graphemes may be more susceptible to migration than others.

## CONCLUSION

The aim of this multiple single case study was to both replicate and extend previous findings regarding the locus of impairment in English LPD. Our findings converge with previous research by strongly suggesting that LPD cannot be attributed to a phonological output buffer or orthographic input lexicon deficit. Rather, our results suggest that LPD is most likely caused by a deficit specific to the coding of letter position at the orthographic-visual analysis stage of reading.

In line with previous studies, however, there was some variability in performance amongst the three children on the tasks designed to explicitly assess for an orthographic-visual analysis deficit. One thing that is becoming increasingly clear as research on LPD progresses is that localizing the source of the migration errors seen in LPD is no easy feat. While identifying what *does not cause* migration errors (i.e., a phonological output or orthographic input lexicon deficit) is relatively straightforward, identifying what *causes* migration errors is not as clear-cut. The findings from the present study suggest that variations in the manifestation of an orthographic-visual analysis deficit may be, at least in part, due to individual differences in strategy use. Therefore, to maximize the potential of localizing the deficit underpinning LPD, future research needs to ensure that the tasks used either eliminate or greatly reduce the opportunity for compensatory strategies to be adopted.

Finally, the finding that the three children in the present study were found to have great difficulty in reading migratable words, in the absence of any other obvious reading or spoken language difficulty, attests to the heterogeneity of dyslexia and its underlying causes. Our findings strongly suggest that not all children with reading difficulties have an impairment in phonological processing. Rather, our findings join a growing body of research in advocating the need to map this heterogeneity in developmental dyslexia, and to develop diagnostic tools that assess the variety of its underlying causes.

## Conflict of Interest Statement

The authors declare that the research was conducted in the absence of any commercial or financial relationships that could be construed as a potential conflict of interest.

## Footnotes

1 Homeschooling for LM followed a strict and regulated curriculum matched to mainstream education. The work completed by home-schooled students has to be documented and monitored regularly.

2 Note that there are currently no normative data published for children who are LL’s age (11 years 9 months). LL’s performance on the CC2, as well as that of her control group, was therefore compared to the normative data of children between the ages 11 years and 11 years 5 months.

3 The task was designed to have 140 word pairs and 140 consonant–string pairs. However, one word pair in the same migration condition (e.g., *slime–slime*) and one consonant–string pair in the different migration condition (e.g., *dktlp–dltkp)* were removed from the analysis as they were not presented correctly.

4 Note that following this finding we also analysed the influence of internal bigram frequency on migration errors using Solso and Juel’s (1980) bigram frequency count database. That is, we analyzed whether or not LM and LL were more likely to migrate the lower frequency bigram *li* in the non-word *p****li****m* into the higher frequency bigram *il* (resulting in the misreading of the non-word as “p**il**m”). We found no influence of internal bigram frequency on LM and LL’s migration errors.
